# The SGLT2i “canagliflozin” and the DPP-4i “sitagliptin” mitigate hypertensive nephropathy in adult male rats by modulating the Ang II/RAGE/Nox4/NLRP3 cascade

**DOI:** 10.3389/fphys.2026.1885144

**Published:** 2026-06-23

**Authors:** Fatma E. Hassan, Fatma Al-Zahraa Nabil Al-Shahed, Asmaa Selmy, Lamiaa Mohamed Mahmoud, Basant A. Aldreny

**Affiliations:** 1 Medical Physiology Department, Kasr Alainy, Faculty of Medicine, Cairo University, Giza, Egypt; 2 Department of Physiology, General Medicine Practice Program, Batterjee Medical College, Jeddah, Saudi Arabia; 3 Histology and Cell Biology Department, Faculty of Medicine for Girls, Al-Azhar University, Cairo, Egypt

**Keywords:** angiotensin II, canagliflozin, DPP-4i, hypertension, nephropathy, RAGE, SGLT2i, sitagliptin

## Abstract

**Background:**

Hypertensive nephropathy (HN) with progressive renal damage is a common consequence of arterial hypertension (HTN). This study addresses the renoprotective attributes of the sodium-glucose cotransporter-2 inhibitor canagliflozin (Cana) and the dipeptidyl peptidase-4 inhibitor sitagliptin (Sita) on HN.

**Methods:**

Twenty-four adult male Wistar rats were categorized into four groups (six per group): control (CTRL), hypertensive nephropathy (HN); rats were given L-NAME (50 mg/kg, i.p., once a day), HN + Cana; rats were given L-NAME injections alongside oral Cana 10 mg/kg, and HN + Sita; rats were provided Sita 10 mg/kg concurrently with L-NAME injection. All regimens were given once a day for five consecutive weeks. Various physiological, biochemical, molecular, and histological parameters were evaluated.

**Results:**

Administration of Cana and Sita alleviated HN, as demonstrated by the notable improvements in renal functions, plasma angiotensin II, and systolic blood pressure. Additionally, a noticeable improvement in renal superoxide dismutase, malondialdehyde, NOD-like receptor family pyrin domain containing 3 inflammasome, interleukin (IL)-1β/-10, caspase 1, advanced glycation end products (AGEs), and relative expression of receptors for AGEs and nicotinamide adenine dinucleotide phosphate oxidase 4 was detected. Substantial enhancement in the microscopic structure of renal tissues, attenuated renal fibrosis, and decreased immunoreactivity of BAX and tumor necrosis factor-α highlighted these protective attributes.

**Conclusion:**

Both Sita and Cana successfully attenuated HTN and subsequent HN, thereby restoring renal function; however, Sita’s protective profile was more favorable. These results implied that Sita and Cana might provide renoprotective impacts for kidney damage triggered by HTN in rats. However, additional research to investigate their possible modes of action is necessary.

## 1 Introduction

Arterial hypertension (HTN) is among the top leading causes of death globally (O’Connell et al., 2026). Chronic uncontrolled HTN has a substantial influence on several organs, including the kidneys, which may culminate in hypertensive nephropathy (HN) with subsequent end-stage renal disease (Smith and Taubert, 2025; Suarez et al., 2025).

N^ω^-nitro-l-arginine methyl ester hydrochloride (L-NAME) hinders nitric oxide synthase (NOS) and increases angiotensin II (Ang II) via activating Ang II-converting enzyme (ACE), hence causing vasoconstriction and renal blood flow drop (Rüster et al., 2009).

The tubulointerstitium and glomeruli of HN patients exhibit intense expression of the receptor for advanced glycation end products (RAGE) (Abel et al., 1995). Studies showed that patients with HN accumulate advanced glycation end products (AGEs) (Tanji et al., 2000) and that the kidneys of adult spontaneously hypertensive rats (SHRs) exhibit activation of AGEs/RAGE (Liu et al., 2020). Additional information from cultured podocytes indicates that Ang II-induced activation of the Ang II type 2 receptor (AT2R) leads to enhanced RAGE synthesis (Rüster et al., 2009). Moreover, according to Olopade et al. (2024), Ang II induces oxidative stress (OS) (Olopade et al., 2024), which exacerbates HTN and kidney damage (Xiao et al., 2025).

Intrarenal nicotinamide adenine dinucleotide phosphate (NADPH) concentration surges, and NADPH oxidase (Nox) is activated in renal arterioles, glomerular and tubular cells, macula densa, and podocytes because of reactive oxygen species (ROS) accumulation, reducing nitric oxide (NO) and thereby leading to the emergence/aggravation of HTN (Haidara et al., 2025). Worth noting, Ang II has been shown to increase Nox activity, especially Nox4, in the kidneys (Wang et al., 2024). Additionally, in diabetic and aging renal diseases, activation of AGE-mediated RAGE may trigger Nox-induced ROS generation, ensuing OS (Wu et al., 2021). Thus, identifying therapeutic strategies that may modulate Ang II, RAGE, and OS may offer multifaceted potential in managing HTN and its associated HN.

A novel category of antihyperglycemic medications called canagliflozin (Cana) lowers blood sugar by inhibiting sodium-glucose cotransporter 2 (SGLT2) in proximal convoluted tubules (PCTs). Also, Cana enhances arterial flexibility and attenuates HTN and inflammation. Additionally, SGLT2 inhibitors (SGLT2i) demonstrated advantageous renal functions (El Khayari et al., 2024); Cana reduces kidney damage and HTN by preventing intrarenal angiotensinogen activation (Woods et al., 2019) and minimizes liver damage by modifying RAGE signaling (Abdelmageed and Abdelrahman, 2023).

A selective inhibitor of dipeptidyl peptidase-4 (DPP-4i) is sitagliptin (Sita). Apart from its ability to decrease blood sugar, it has many other positive impacts without resulting in hypoglycemia in normoglycemic animals (Arab et al., 2023; Hassan et al., 2026). In experimental chronic renal disease, Sita lowers cardiac Ang II (Beraldo et al., 2019) and reduces arterial calcification by inhibiting OS-induced RAGE (Lin et al., 2021).

Evaluating and contrasting an SGLT2i with a DPP-4i in a non-diabetic model offers distinct pathophysiological perspectives, even though both agents are primary glucose-lowering medications. This enables us to recognize and fully grasp their direct, glucose-independent renoprotective mechanisms, particularly how they affect inflammatory cascades, intrarenal OS, and hemodynamic forces distinctively under extreme hypertensive stress.

Given the advantages of either Cana or Sita, we aimed to evaluate their renoprotective effects on HTN-induced renal damage. Specifically, we investigated their potential on the inflammation, apoptosis, and Ang II/RAGE/Nox4-driven OS in the kidneys, which might offer a prospective solution to a major health issue with a greater socioeconomic burden that HTN presents.

## 2 Materials and methods

### 2.1 Ethical approval

To improve transparency and reproducibility, this experiment was conducted in accordance with the ARRIVE standards, the U.K. Animals (Scientific Procedures) Act 1986 and associated guidelines, EU Directive 2010/63/EU for animal experiments, and the National Institutes of Health Guide for the Care and Use of Laboratory Animals (NIH Publications No. 8023, revised 1978). In addition, this study was approved by the Cairo Institutional Animal Care and Use Committee (IACUC), Cairo University, Egypt (CU III F 6 25).

### 2.2 Drugs

L-NAME, canagliflozin, and sitagliptin were supplied by Sigma-Aldrich (USA), Janssen Pharmaceutical Co. (Titusville, NJ, USA), and Adamas Reagent Co. (Shanghai, China), respectively.

### 2.3 Study design and animal procedures

Twenty-four fully grown (225 ± 10 g) male Wistar rats were used in this experiment, which was conducted in the Cairo University’s Faculty of Medicine animal facility in Egypt. Rats were kept three per cage with well-ventilated covers at an ambient temperature (25 °C ± 5 °C) and under a standard light/dark cycle. Water and laboratory rat food were provided *ad libitum* throughout the study. The sample size was determined *a priori* using G*Power 3.1.9.7 for one-way ANOVA (fixed effects, omnibus), based on an effect size *f* = 0.78809 (Bishr et al., 2025), alpha = 0.05, and power = 0.80, which yielded a total sample size of 24 rats (*n* = 6 per group).

Before the research study began, rats were housed for 7 days to acclimatize to the experimental conditions. They were subsequently assigned to four groups using a simple randomization procedure (*n* = 6): control (CTRL), rats received both oral and intraperitoneal (i.p.) normal saline; HN group, rats were given L-NAME (50 mg/kg, i.p., once a day) (Hassan et al., 2024); HN + Cana group, rats were provided with L-NAME injections together with oral Cana 10 mg/kg (Bishr et al., 2025); and HN + Sita group, rats were administered Sita 10 mg/kg concomitantly with L-NAME injection (Arab et al., 2021). All therapeutic regimens were given once a day for five consecutive weeks. Systolic blood pressure (SBP) was recorded 24 hours after the experimental trial concluded. Urine volume was quantified after being gathered from the metabolic cages. Then, blood samples were collected under anesthesia using ketamine/xylazine (60/6 mg/kg) (Abbas et al., 2022). Immediately after, cervical dislocation was performed followed by kidney dissection and extraction.

The study was designed to compare the effects of canagliflozin and sitagliptin against the HN condition; therefore, single-drug-only groups and an ACE inhibitor (ACEI)/angiotensin receptor blocker (ARB) comparator were not included in this exploratory design to focus on treatment-versus (vs.)-disease comparisons.

### 2.4 Measured variables

SBP measurement and biochemical, molecular, histological, and immunohistochemical (IHC) investigations were carried out blindly, disregarding the experimental group assignment, to ensure objectivity and minimize bias.

#### 2.4.1 Physiological variables

##### 2.4.1.1 SBP

SBP was measured in conscious rats using a non-invasive tail-cuff blood pressure device (ML 125 NIBP, AD instruments Pty. Ltd., Sydney, Australia) after acclimatization to the procedure. Measurements were obtained under standardized conditions at the same time of the day, and multiple readings were taken for each animal to obtain a representative value.

##### 2.4.1.2 Urine flow

Urine flow (mL/min) =Urine volume (24 h)/1,440  (Hassan et al., 2023).

#### 2.4.2 Chemical and molecular parameters

All biochemical assays were performed according to the manufacturer’s instructions, and samples from all groups were analyzed in the same run whenever possible to minimize interassay variability.

##### 2.4.2.1 Plasma Ang II

Plasma levels of Ang II were assayed by the Ang I ELISA Kit (Cat# E-EL-R1430, Elabscience, Houston, TX, USA).

##### 2.4.2.2 Serum blood urea nitrogen, serum creatinine, and urinary creatinine

Serum blood urea nitrogen (BUN) was measured by the BUN ELISA kit (Cat# MBS2611086, MyBioSource, CA, USA). Serum creatinine (Scr) and urinary creatinine (Cr) were measured by the Cr Assay Kit (Cat# ab65340, Abcam, Waltham, MA, USA).

##### 2.4.2.3 Glomerular filtration rate


Cr clearance(mL/min)=Urine Cr(mg/dL)×Urine flow(mL/min)/Scr(mg/dL)

##### 2.4.2.4 Renal tissue oxidant/antioxidant parameters

Renal tissue superoxide dismutase (SOD) activity (Cat# 7500-100-K; Trevigen, Gaithersburg, MD, USA) and malondialdehyde (MDA) (Cat# ab233471) were evaluated colorimetrically.

##### 2.4.2.5 Renal tissue AGEs, NLRP3, IL-1β, IL-10, and caspase 1

After homogenizing and washing in phosphate-buffered saline, the right kidneys were stored overnight at −20 °C. After the cell membranes were broken down by two freeze–thaw cycles, the homogenate was centrifuged at 5,000×*g* for 5 min at 4 °C. We extracted the supernatant and used the following ELISA kits for each renal tissue sample: Cat# ab238539 (Abcam) to assess AGEs concentration, Cat# ab277086 to detect NOD-like receptor family pyrin domain containing 3 (NLRP3) level colorimetrically, Cat# MBS2023030 (MyBioSource) to measure interleukin-1β (IL-1β), Cat# ab100765 to quantify interleukin-10 (IL-10) and Cat# MBS451267 (MyBioSource) to detect caspase 1.

##### 2.4.2.6 Quantitative real-time polymerase chain reaction of renal tissue RAGE and Nox4

Total RNA was extracted from kidney tissue using the RNX-Plus reagent (CinnaGen, Iran). One microgram of total RNA was then reverse-transcribed into single-strand cDNA using the RevertAid™ First Strand cDNA synthesis kit (Thermo Scientific, Lithuania). Next, quantitative real-time polymerase chain reaction was performed using a LightCycler^®^ 96 System (Roche Life Science, Deutschland GmbH Sandhofer, Germany) and RealQ Plus 2x Master Mix Green (Ampliqon, Denmark). The amplification technique involved one cycle at 95 °C for 15 min, followed by 40 cycles at 95 °C for 20 s, 58 °C for 30 s, and 72 °C for 30 s. The specificity of amplification was verified by primer design and melting curve analysis, and relative gene expression of all groups was calculated using the 2^−ΔΔCt^ method with β-actin as the internal reference gene (Liu et al., 2020). The genes to be studied were RAGE and Nox4. Primer sequences were as follows: *RAGE*, forward: 5′-GAGTCCGAGTCTACCAGATTCC-3′, reverse: 5′-GGTCTCCTCCTTCACAACTGTC-3′; *Nox4*, forward: 5′-GAACCTCAACTGCAGCCTGATC-3′, reverse: 5′-CTTTTGTCCAACAATCTTCTTGTTCTC-3′; and *β-actin*, forward: 5′-ATCAGCAAGCAGGAGTACGAT-3′, reverse: 5′-AAAGGGTGTAAAACGCAGCTC-3′.

#### 2.4.3 Histopathological assessment of the kidneys

##### 2.4.3.1 Light microscopy examination

Kidney sections were preserved in formalin (10%) then cut into sections (5 µm) for light microscopy (LM) examination (Suvarna et al., 2018; Hassan et al., 2023). Hematoxylin and Eosin (H&E) stain was used to examine the general structure of the kidneys. Further, Masson trichrome (MT) stain was performed to study the distribution and amount of collagen fibers in kidney tissues. (Kiernan, 2015).

##### 2.4.3.2 Immunohistochemical staining

To study the extent of renal tissue apoptosis and inflammation, IHC stains for BAX (Kiernan, 2015) and tumor necrosis factor-alpha (TNF-α) (Jammal et al., 2015) were utilized.

##### 2.4.3.3 Histomorphometry studies

Ten randomly selected non-overlapping fields were subjected to a quantitative analysis of area percent (%) at a fixed measuring magnification of (×400) to detect the distribution of collagen fibers, BAX expression, and TNF-α expression in renal cortical tissue utilizing a Leica QWin V3 image analysis software (Leica Microsystems, Wetzlar, Germany) on a computerized morphometry system coupled to a digital camera-mounted microscope. The image analyzer was first calibrated automatically to convert the measurement units (pixels) produced by the image analyzer program into actual micrometer units.

### 2.5 Statistical analysis

SPSS 26 was used to process data. The Shapiro–Wilk test was used to verify the normal distribution of the data. To assess differences between groups, Tukey’s *post hoc* test for multiple comparisons was applied in combination with one-way analysis of variance (ANOVA). The outcomes were then shown as mean ± standard deviation. The significance level is *p ≤*0.05. Unless *p* < 0.001 (stated as *p* < 0.001), precise *p*-values were mentioned.

## 3 Results

### 3.1 Physiological, biochemical, and molecular results

#### 3.1.1 SBP

As displayed in **Table 1**, SBP was significantly elevated in the HN group. Despite the SBP being greatly improved on either treatment with Cana or Sita, it did not reach CTRL values. Nevertheless, within this model, Sita’s effect was significantly more superior to that of Cana.

**Table 1:** SBP, serum Ag II, and KFT results.

Parameter	CTRL	HN	HN + Cana	HN + Sita
SBP (mm Hg)	114.17 ± 5.85	180.00 ± 8.94* *p < 0.001	153.33 ± 8.17*$ *$*p* < 0.001	135.83 ± 5.85*$# *$*p* < 0.001 #*p* = 0.003
Plasma Ang II (pg/mL)	7.67 ± 0.87	16.83 ± 2.32* *p < 0.001	10.87 ± 0.98*$ *p = 0.004 $*p* = <0.001	10.80 ± 0.75*$ *p = 0.004 $*p* = < 0.001
Scr (mg/dL)	1.11 ± 0.13	2.52 ± 0.45* *p < 0.001	1.84 ± 0.12*$ *p = 0.001 $*p* < 0.001	1.54 ± 0.20*$ *p = 0.043 $*p* < 0.001
Serum BUN (mg/dL)	21.67 ± 1.63	33.17 ± 4.07* *p < 0.001	27.00 ± 1.67*$ *p = 0.005 $*p* = 0.001	25.83 ± 1.17*$ *p = 0.034 $*p* < 0.001
Urine volume (mL/24 h)	11.08 ± 0.78	4.08 ± 1.11* *p < 0.001	6.67 ± 0.45*$ *$*p* < 0.001	8.483 ± 0.17*$# *$*p* < 0.001 #*p* = 0.002
Urine flow (mL/min)	0.0081 ± <0.0015	0.0028 ± <0.0018* *p < 0.001	0.0046 ± <0.0013*$ *$*p* < 0.001	0.0059 ± <0.0011*$# *$*p* < 0.001 #*p* = 0.002
Urinary creatinine (mg/dL)	119.17 ± 7.52	74.38 ± 13.71* *p < 0.001	106.68 ± 5.93$ $*p* < 0.001	116.77 ± 6.80$ $*p* < 0.001
GFR (mL/min)	0.83 ± 0.07	0.09 ± 0.04* *p < 0.001	0.27 ± 0.01*$ *$*p* < 0.001	0.45 ± 0.05*$# *$#*p* < 0.001

Data are presented as mean ± standard deviation. CTRL, control; HN, hypertensive nephropathy; Cana, canagliflozin; Sita, sitagliptin; SBP, systolic blood pressure; KFTs, kidney function tests; Scr, serum creatinine; BUN, blood urea nitrogen; Ang II, angiotensin II; GFR, glomerular filtration rate.

*: Significant vs. CTRL, ^$^significant vs. HN, ^#^significant vs. HN + Cana. *p ≤*0.05 means statistically significant.

#### 3.1.2 Kidney function test results

Significant deteriorations in kidney function tests (KFTs) [Scr, serum BUN, urine volume, urine flow, urine Cr, and glomerular filtration rate (GFR)] were noticed in the HN group as shown in **Table 1**. Nevertheless, these parameters were significantly and comparably improved on either Cana or Sita treatment; however, they did not reach baseline (CTRL) levels except for the urinary Cr of the Sita group.

#### 3.1.3 Plasma Ang II

A significant rise in plasma Ag II was noticed in the HN group as shown in **Table 1**. Nevertheless, its level was significantly and comparably decreased after treatment with either Cana or Sita, without reaching the standard levels.

#### 3.1.4 Renal AGEs content

The AGEs significantly (*p* < 0.001) increased in the HN group’s renal tissues. Relative to HN’s values, AGEs considerably decreased (although they did not reach CTRL values; *p* < 0.001) in the Cana (*p* = 0.004) and Sita (*p* < 0.001) groups. Worth noting, the Sita effect on renal AGEs was superior to that of Cana (*p* = 0.004) (**Figure 1**).

**Figure 1:**
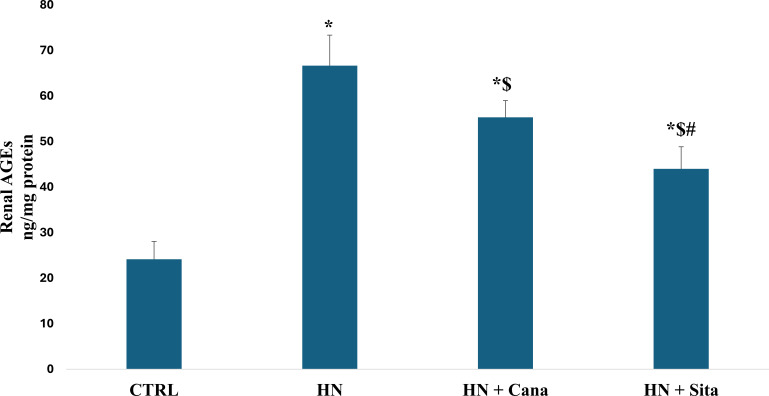
Renal AGEs content. AGEs, advanced glycation end products; CTRL, control; HN, hypertensive nephropathy; Cana, canagliflozin; Sita, sitagliptin. Data displayed as means ± SD. *Significant vs. CTRL, ^$^significant vs. HN, ^#^significant vs. HN + Cana. *p ≤*0.05 implies statistical significance.

#### 3.1.5 Renal *RAGE* and *Nox4* gene expression

As revealed in **Figure 2**, the renal *RAGE* and *Nox4* mRNA gene expression was substantially amplified (*p* < 0.001) in the HN group compared to the CTRL. Renal *RAGE* and *Nox4* gene expression was significantly improved in the Cana (*p* = 0.004 and *p* < 0.001, respectively) and Sita (*p* < 0.001) groups relative to the HN group. However, Sita’s impact was more noticeable (*p* < 0.001) on *Nox4* mRNA gene expression when compared to that of Cana.

**Figure 2:**
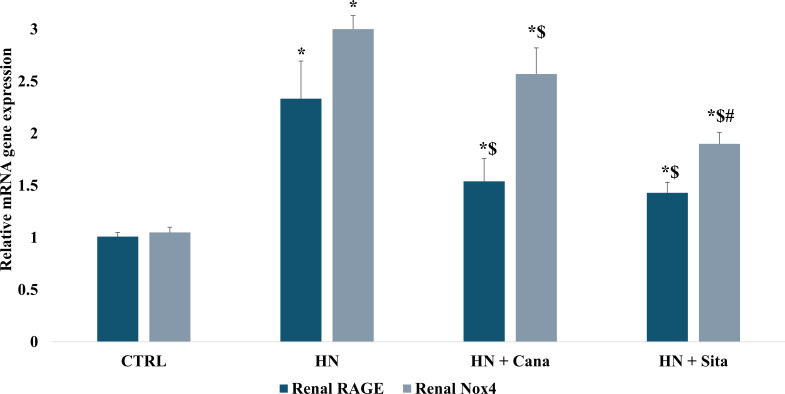
Renal *RAGE* and *Nox4* gene expression. RAGE, receptor for advanced glycation end products; Nox4, NADPH oxidase 4; CTRL, control; HN, hypertensive nephropathy; Cana, canagliflozin; Sita, sitagliptin. Data displayed as means ± SD. *Significant vs. CTRL, ^$^significant vs. HN, ^#^significant vs. HN + Cana. *p ≤*0.05 implies statistical significance.

#### 3.1.6 Renal SOD and MDA

As displayed in **Figure 3**, the renal OS homeostasis was extensively (*p* < 0.001) disturbed in the HN group (decreased SOD and increased MDA) compared to CTRL values. However, both renal SOD and MDA were significantly improved after treatment with Cana (*p* < 0.001 and *p* = 0.004, respectively) and Sita (*p* < 0.001). Sita achieved a more favorable (*p* = 0.009) effect on SOD than Cana. Yet, compared to CTRL values, the standard levels were neither reached in the Cana nor in the Sita groups (*p* < 0.001).

**Figure 3:**
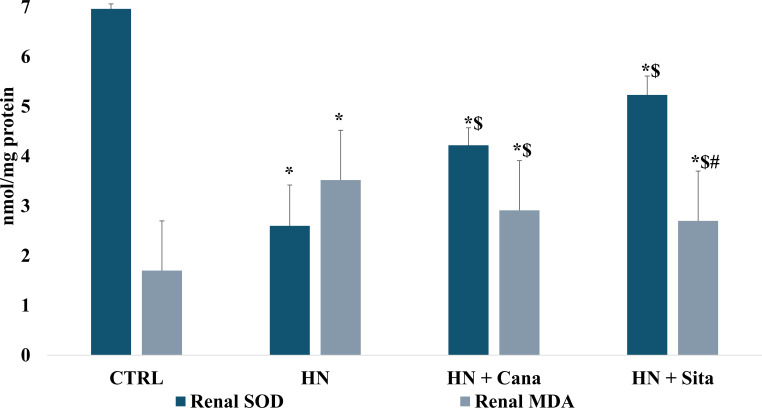
Renal SOD and MDA. SOD, superoxide dismutase; MDA, malondialdehyde; CTRL, control; HN, hypertensive nephropathy; Cana, canagliflozin; Sita, sitagliptin. Data displayed as means ± SD. *Significant vs. CTRL, ^$^significant vs. HN, ^#^significant vs. HN + Cana. *p ≤*0.05 implies statistical significance.

#### 3.1.7 Renal NLRP3, IL-10, and IL-1β

As revealed in **Figure 4**, biochemical analysis of renal tissues showed a significantly elevated NLRP3 and IL-1β concomitant with decreased IL-10 in the HN group vs. CTRL values (*p* < 0.001). In comparison to the HN group, the NLRP3 and IL-1β were considerably lowered, while IL-10 was significantly increased in the Cana- (*p* < 0.001, *p* < 0.001, and *p* = 0.024, respectively) and Sita-treated groups (*p* < 0.001) but did not reach the CTRL values of the Cana group (*p* < 0.001, *p* < 0.001 and *p* = 0.041, respectively) and the Sita group (*p* < 0.001 regarding both NLRP3 and IL-1β), except for the IL-10, which was significantly improved, achieving a relatively normal level. Worth noting, the improvement in both IL-1β and IL-10 had greater significance in the HN + Sita group than in the HN + Cana group (*p* < 0.001).

**Figure 4:**
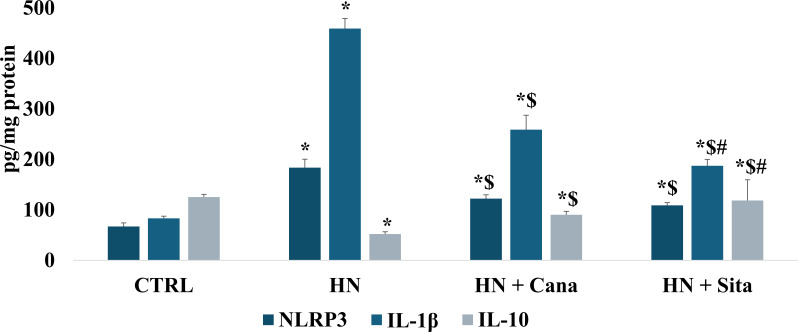
Renal NLRP3, IL-1β, and IL-10. NLRP3, NLR family pyrin domain containing 3; IL-1β, interleukin-1β; IL-10, interleukin-10; CTRL, control; HN, hypertensive nephropathy; Cana, canagliflozin; Sita, sitagliptin. Data displayed as means ± SD. *Significant vs. CTRL, ^$^significant vs. HN, ^#^significant vs. HN + Cana. *p ≤*0.05 implies statistical significance.

#### 3.1.8 Renal caspase 1

As displayed in **Figure 5**, in contrast to CTRL, renal caspase 1 was dramatically elevated in the HN group (*p* < 0.001). However, a significant decline in caspase 1 in either the Cana or Sita groups (*p* < 0.001) with a more superior improvement reported in the HN + Sita group (*p* = 0.014) was detected, in contrast to the Cana group. However, normal CTRL values were not reached with both treatment regimens (*p* < 0.001).

**Figure 5:**
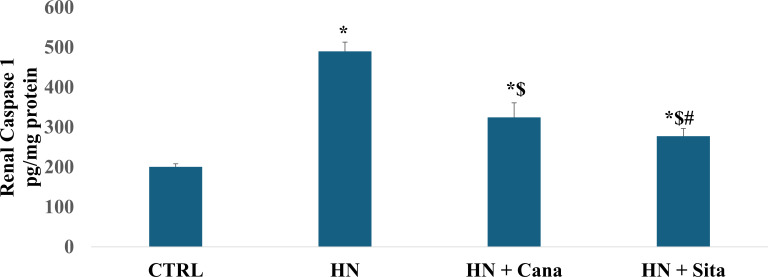
Renal caspase 1. CTRL, control; HN, hypertensive nephropathy; Cana, canagliflozin; Sita, sitagliptin. Data displayed as means ± SD. *Significant vs. CTRL, ^$^significant vs. HN, ^#^significant vs. HN + Cana. *p ≤*0.05 implies statistical significance.

### 3.2 Histopathological examination results

#### 3.2.1 H&E results

LM examination of CTRL kidneys showed a typical histological architecture, comprising the glomerulus (G), Bowman’s capsule (B), and PCTs (P) exhibited a narrow lumen which is lined by high cuboidal epithelium with deep acidophilic cytoplasm and central rounded nuclei with prominent brush border, in contrast to distal convoluted tubules (D), which showed wide lumen and lined by low cuboidal cells with a pale acidophilic cytoplasm and rounded nuclei missing brush border (**Figure 6A**).

**Figure 6:**
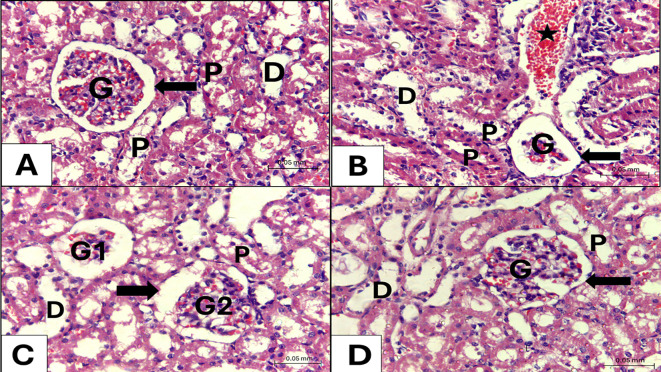
Hematoxylin and eosin staining displaying **(A)** a typical configuration of the renal cortex, glomerulus (G), Bowman’s capsule (black arrow), proximal convoluted tubule (P), and distal convoluted tubule (D); **(B)** shrunken fibrotic glomerulus (G), Bowman’s capsule, dilated proximal convoluted tubule with destructed epithelium and brush border (P), area of congested blood vessels (asterisk), and distal convoluted tubule (D); **(C)** some nearly healthy glomerulus (G1), some still shrunken glomerulus (G2), proximal convoluted tubules with brush border (P), dilated Bowman’s space (black arrow), and distal convoluted tubule **(D)**; and **(D)** healthy glomerulus (G), Bowman’s space (black arrow), proximal convoluted tubule (P), and distal convoluted tubule **(D)**. H&E ×400.

Examination of the HN’s kidneys revealed shrunken, atrophic glomeruli with a shrunken whole Malpighian renal corpuscle with dilatation of Bowman’s space. Regarding the tubules, there was severe atrophy of the renal tubules, mainly PCTs, manifested as loss of brush border, destruction of the epithelium, and dilatation of the lumen (**Figure 6B**).

Examination of the HN + Cana’s kidneys revealed a slight improvement in the microscopic structure of the kidney; some Malpighian renal corpuscles showed an increase in size and narrowing in Bowman’s space in comparison to the HN group. PCTs showed improvement in brush border, lumen size, and epithelium (**Figure 6C**).

Examination of the HN + Sita’s renal tissues showed better improvement in the microscopic structure of the kidney to be near normal. Malpighian renal corpuscle revealed an increase in size, Bowman’s space narrowing, and improvement in glomeruli architecture. The tubules showed increased brush border, lumen size, and epithelium (**Figure 6D**).

#### 3.2.2 MT stain results

Examination of the CTRL group revealed scanty collagen fibers (stained in green) around Malpighian renal corpuscles, around tubules, and in between tubules and corpuscles (**Figure 7A**), whereas examination of the HN group revealed prominent proliferation in collagen fibers surrounding corpuscles, tubules, and the areas between them (**Figure 7B**). Treatment with Cana led to a slight decrease in collagen fibers (**Figure 7C**), while treatment with Sita caused an obvious decrease in collagen fibers (**Figure 7D**).

**Figure 7:**
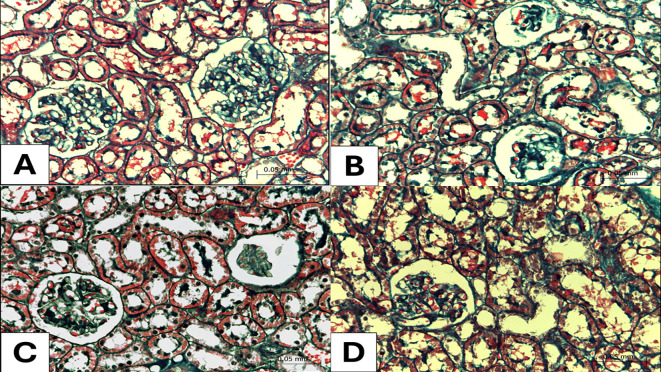
Masson trichrome staining showing **(A)** minimal collagen fibers around Malpighian renal corpuscle and tubules (stained green), **(B)** increased collagen fibers around the renal corpuscle and tubules and in interstitial spaces between tubules, **(C)** slightly decreased collagen fibers around renal corpuscle and tubules, and **(D)** markedly decreased collagen fibers around renal corpuscle and tubules. Masson trichrome ×400.

#### 3.2.3 IHC results

IHC staining revealed a minimal amount and distribution of apoptotic cells by the BAX IHC stain, which appeared as intracytoplasmic brown spots in the CTRL group (**Figure 8A**). Examination of the HN group showed a marked increase in the BAX IHC reaction (**Figure 8B**). In the Cana-treated group, the BAX IHC reactions showed a minimal decrease in comparison to the HN group (**Figure 8C**), while in the Sita-treated group, there was a marked decrease in BAX reactions (**Figure 8D**).

**Figure 8:**
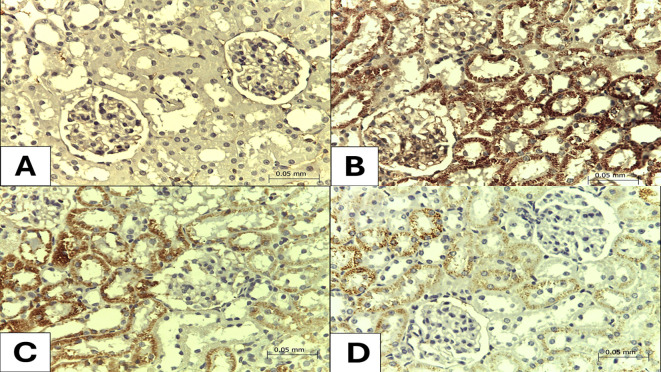
BAX immunohistochemical reaction showing **(A)** scanty +ve BAX immunohistochemical reaction in the control group (brown spots), **(B)** strongly +ve BAX immunohistochemical reaction in glomerulus CTRL tubules, **(C)** slightly reduced BAX reaction in relation to HN, and **(D)** markedly reduced BAX reaction in relation to HN. BAX ×400.

TNF-α +ve reactions also appeared as intracytoplasmic brown spots. It was minimally noticed in the CTRL group (**Figure 9A**). Examination of HN renal tissues showed a marked increase in the TNF-α reactions (**Figure 9B**). In the HN + Cana group, TNF-α IHC reactions showed minimal decrease in comparison to HN (**Figure 9C**), while in the HN + Sita group, there was a marked decrease in TNF-α reaction (**Figure 9D**).

**Figure 9:**
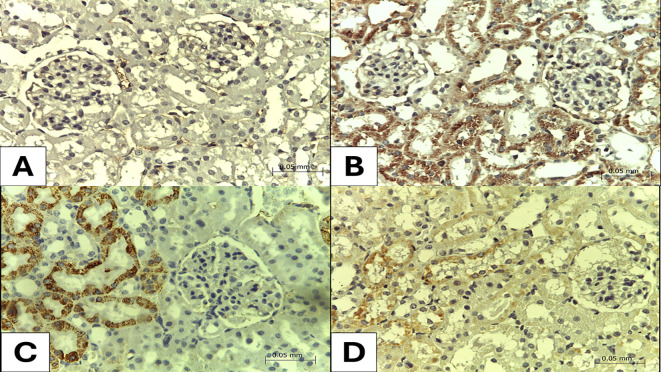
TNF-α immunohistochemical reaction showing **(A)** showing minimal +ve reaction to TNF-α, **(B)** strong +ve reaction to TNF-α, **(C)** slightly decreased reaction to TNF-α in comparison to HN, and **(D)** obviously decreased reaction to TNF-α when compared to HN. TNF-α ×400.

#### 3.2.4 Histomorphometry results

As depicted in **Figure 10**, the kidneys of the HN group exhibited a notable increase in collagen fibers area % as opposed to CTRL. The HN + Cana group displayed a slight decline in the area % of collagen fibers, which was statistically significant relative to both CTRL and HN groups. Meanwhile, the HN + Sita group exhibited an obvious decrease in collagen area %, which was also statistically sizable vs. the other experimental groups.

**Figure 10:**
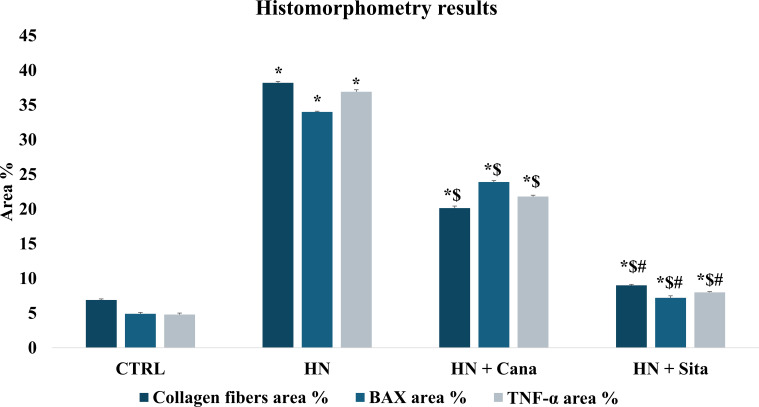
Area percentage of renal collagen, BAX, and TNF-α in renal tissues. TNF-α, tumor necrosis factor-α; CTRL, control; HN, hypertensive nephropathy; Cana, canagliflozin; Sita, sitagliptin. Data displayed as means ± SD. *Significant vs. CTRL, ^$^significant vs. HN, ^#^significant vs. HN + Cana. *p ≤*0.05 implies statistical significance (one-way ANOVA followed by Tukey’s multiple comparison test).

Similarly, a significant rise in BAX and TNF-α + ve cells area % in the HN group, relative to the CTRL group, was observed. However, administration of Cana caused a slight decrease in area % of both BAX and TNF-α +ve cells, which was statistically significant in comparison to the CTRL and HN groups. Meanwhile, administration of Sita caused a marked decrease in area % of BAX as well as TNF-α + ve cells, which was statistically significant in comparison to the other experimental groups (*p* < 0.001).

## 4 Discussion

The administration of sitagliptin and canagliflozin both alleviated HN, as demonstrated by the notable improvements in renal functions, plasma Ang II, and SBP. Additionally, the consumption of sitagliptin and canagliflozin was linked to a noticeable improvement in renal OS homeostasis (increased SOD and decreased MDA), inflammatory biomarkers (increased IL-10 and decreased NLRP3 and IL-1β), caspase 1 and AGEs, and relative expression of RAGE and Nox4. The substantial enhancement in the microscopic structure of renal tissues, decreased renal fibrosis, and decreased immunoreaction of the apoptotic (BAX) and inflammatory (TNF-α) markers highlighted these protective attributes.

### 4.1 Nephrotoxic effects accompanying HTN

In L-NAME-induced HTN, elevated ACE activity causes Ang II to directly constrict renal blood vessels, increasing mechanical stress to the glomerulus and mesangial cells by cytokines, e.g., TNF-α, that restrict renal blood flow. HN is also triggered and aggravated by diminished NOS expression as well as augmented OS and Nox (Alsuwayt, 2025). By raising NADPH levels and activating Nox, which lowers NO bioavailability, ROS are created in the kidneys’ arterioles, glomerular/tubular cells, macula densa, and podocytes. These ROS do not merely contribute to the onset of HTN but also to the immunological, renal, and renin–angiotensin system (RAS) cellular mechanisms that lead to its consequences, such as HN (Arendshorst et al., 2024).

Research revealed that Ang II-induced activation of the AT2R in podocytes led to a rise in RAGE generation (Rüster et al., 2009). Additionally, it has been shown that AGEs accumulation is expedited and enhanced in hypertensive patients, where there is a substantial correlation between plasma AGEs and aortic stiffness. This was explained by the decreased NO bioavailability, endothelin-1 induction, and interaction with RAGEs, which are significantly expressed in the activated vascular endothelium (Geroldi et al., 2005). Furthermore, by attaching to lipoproteins, plasma AGEs can inhibit their elimination. This relationship could foster inflammation and atheromatous plaque development (Veiraiah, 2005).

Another study found that elevated plasma AGEs in hypertensive individuals can be caused by lower plasma RAGE levels, which could help remove or detoxify plasma AGEs by serving as decoys (Geroldi et al., 2005). The interaction between AGEs and RAGE causes OS and promotes inflammatory and fibrotic responses in the kidneys, leading to an eventual deterioration in renal morphology and function (Yamagishi et al., 2008; Manigrasso et al., 2014).

Likewise, ROS promote kidney inflammation and organ damage by converting procaspase to active caspase, which in turn changes the inactive pro-IL-1β into its active variant. Moreover, the NLRP3 inflammasome pathway may be further triggered by this OS, which could result in the advancement of HN. In addition to regulating the synthesis and evolution of pro-inflammatory cytokines like IL-1β, the NLRP3 inflammasome also regulates caspase 1 activation, which injures renal podocytes and results in nephrin loss. It also alters the expression of elastic collagen membranes, which aggravates albuminuria. Furthermore, podocin and nephrin malfunctions contribute to HTN-induced glomerulosclerosis and nephropathy (Alsuwayt, 2025).

At the microscopic level, examination of the untreated group kidneys revealed almost the main manifestations of HN, which is in agreement with previous studies (Ray and Reddy, 2023; Nagata and Hishida, 2024; Zeng and Yang, 2024). These changes were most probably caused by narrowing of blood vessels, mainly afferent arterioles, leading to different grades of sclerosis of the Malpighian renal corpuscle and destruction of the tubules (Renna et al., 2013; Wenzel et al., 2015). The increase in TNF-α as well as the apoptotic marker (BAX) was explained by the enhanced inflammatory reactions in renal disorders mainly in the corpuscles and tubules, which lead to increased apoptosis of the cells lining tubules and glomeruli. This was also accompanied by fibrosis of different parts of the renal cortex (Hanaoka et al., 2025; Hubbi et al., 2025), which explains the increase in fibrous tissue by the MT stain in the HN group.

Therefore, given that HN is the second most prevalent contributor of end-stage renal disease globally (Lai et al., 2025), it is imperative to discover effective treatments for this condition. Significant morbidity, mortality, and healthcare expenditures are caused by this emergence, and the burden of chronic renal disease linked to HTN is expected to rise steadily. Since current approaches frequently fail despite strict arterial blood pressure (ABP) control, new treatments are required to halt the progression of HN.

### 4.2 Renoprotective effects of canagliflozin against HN

The most prevalent pharmacological treatments for halting the course of renal disease are RAS inhibitors. Nevertheless, novel medications were associated with favorable results for kidney health. SGLT2i is one of these newly developed therapies. Apart from its effect on blood glucose, SGLT2i has cardiovascular and renal beneficial effects. It causes glucosuria and natriuresis, leading to hypovolemia. More sodium delivery to the distal convoluted tubule will stimulate the macula densa, resulting in afferent arteriole constriction, lowering intraglomerular pressure and glomerular perfusion. But as time passes, the amount of Ang II falls, which leads to a drop in inflammation and OS and a rise in intrarenal oxygenation, both of which raise GFR (Perkovic et al., 2019).

No research has been accomplished on how SGLT2i affects AGEs and RAGE in the kidneys of rats without diabetes. But according to earlier studies, SGLT2 knockdown prevents human cultured proximal tubular cells from producing ROS and expressing RAGE when exposed to high glucose. Furthermore, they discovered that SGLT2-dependent glucose absorption into tubular cells in diabetic nephropathy (DN) exacerbated the cells’ sensitivity to pro-apoptotic consequences of AGEs via RAGE overexpression (Maeda et al., 2013; Vallon et al., 2013; Kitada et al., 2014; Fukami et al., 2015).

A further study reported the efficiency of SGLT2i to suppress pro-oxidants like Nox4 (Tahara et al., 2014), reduce the generation of free radicals (Steven et al., 2017), and boost the capacity of antioxidant enzymes (e.g., SOD) (Yaribeygi et al., 2023). Increased ROS generation is further related to increased Nox4 expression in the heart and kidneys, which could be reduced by Cana (Hasan et al., 2020).

In line with the study of Li and his colleagues, SGLT2i dramatically lowers the levels of Nox4 in diabetic rats’ heart tissue, mostly by lowering Nox activity with potential for reversing diabetic cardiomyopathy (Li et al., 2019).

SGLT2i, irrespective of hypoglycemia pathways, may directly impact the pathways of inflammation (Lopaschuk and Verma, 2020). According to a previous research, overweight or obese non-diabetic adults, can safely and well endure SGLT2i (Zheng et al., 2021). Under conventional glucose circumstances, SGLT2i targets NLRP3 expression, OS induced by IL-17A, and inflammatory reactions thereby preventing smooth muscle cells (SMCs) migration and proliferation without causing cell death (Sukhanov et al., 2021).

Additionally, it was found that SGLT2i minimizes renal damage by increasing adenosine 5′-monophosphate-activated protein kinase and sirtuin1 expression while lowering kidney NLRP3, IL-1β, and caspase 1 expression (Zhu et al., 2025).

Also, Cana’s antioxidant influence on the kidneys may be explained by its diuretic action, which may dilute OS indicators in the urine, triggering antioxidant and anti-inflammatory mechanisms, and suppression of mitochondrial complex I, which reduces mitochondrial stress (Thiel et al., 2024). Additionally, via increasing hypoxia-inducible factor 1-alpha, decreasing inflammation, and apoptosis, Cana enhances kidney homeostasis (Rampersad et al., 2020).

NLRP3 activation in podocytes can worsen glomerular damage and podocyte dysfunction caused by various cellular OS and inflammatory reactions (Yau et al., 2022; Li et al., 2025). Previous research discovered that, while at modest concentrations, podocytes exhibit SGLT2 at baseline. On the other hand, podocytes exposed to elevated glucose levels exhibit a substantial increase in SGLT2 and NLRP3 expression, which is efficiently counteracted by SGLT2i (Ziyadeh and Goldfarb, 1991). Moreover, in individuals with type 2 DN, SGLT2i displays an anti-inflammatory impact by lowering blood IL-1β (Wang et al., 2024) and suppression of ROS/NLRP3/caspase 1-mediated NLRP3 production and IL-17A/18 release in aortic SMCs (Sukhanov et al., 2021). Nevertheless, further investigation is still required to ascertain how Cana impacts the kidneys of hypertensive rats.

At the structural and histological levels, administration of Cana in the HN + Cana group caused a slight improvement in kidney structure in terms of H&E staining, as well as slightly decreased fibrosis as shown by the MT stain and a minimal decrease in tissue inflammatory TNF-α concomitant with a trivial decrease in cellular pro-apoptotic BAX as revealed by the IHC examination. These results agreed with the findings of previous studies (Kruger and Valentine, 2020; Mourad et al., 2023). This could be explained by the fact that administration of Cana caused reduction in kidney fibrosis, OS (Kruger and Valentine, 2020), apoptosis, and inflammatory cytokines, and it was also proven to lower the intestinal absorption of uremic toxins (Mourad et al., 2023).

### 4.3 Renoprotective effects of sitagliptin against HN

Clinical studies have indicated that DPP-4i, an antidiabetic medication, could lower the ABP and shows renoprotective effects without reducing blood sugar (Ogawa et al., 2011; Duvnjak and Blaslov, 2016; Hussain et al., 2016; Chen et al., 2018). Via the AT1 receptor, Ang II was reported to activate renal DPP-4 (Aroor et al., 2016). Therefore, it has been demonstrated that DPP-4i reduces the concentrations of circulating Ang II in SHR (Kawase et al., 2016). Furthermore, DPP-4i raised angiotensin levels (1–7), which were linked to potent antioxidant, antihypertrophic, and anti-inflammatory characteristics (Beraldo et al., 2019).

Compatible with our outcomes, DPP-4i enhanced cardiac ACE2 activity and decreased AT1 receptor expression, while it increased the AT2R expression in rats with Ang II-driven cardiac fibrosis (Zhang et al., 2015), thus improving glucose-independent salt and water management, lowering proteinuria, and limiting glomerular and tubular damage (Lim et al., 2015).

Renal tissues are highly susceptible to oxidative and inflammatory insults due to their high polyunsaturated fatty acid concentration (Jalili et al., 2019). Lin and his colleagues claim that by preventing the emergence of ROS and inflammatory cytokines triggered by Nox activation, Sita lowers nuclear factor-kappa B (NF-κB) activation. This in turn attenuates RAGE/S100a12 signaling. Consequently, Sita reduces the constant NF-κB pathway and RAGE signaling interaction (Lin et al., 2021).

Furthermore, DPP-4 was reported as a primary regulator of T lymphocytes, natural killer cells, and mononuclear/macrophage activation and chemotaxis, which release cytokines, adhesion molecules, chemokines, and ROS, disrupting the vascular endothelium’s normal function and making it more susceptible to HTN (da Silva Júnior et al., 2018). By secreting and delivering Ang II through the endogenous RAS, T cells can raise ABP (Hoch et al., 2009). Consequently, DPP-4i administration lowers cytokine synthesis that regulates T lymphocyte proliferation (Schön et al., 1989). It additionally reduces inflammation by inhibiting monocyte and macrophage chemotaxis and activation (Bengsch et al., 2012; Salim et al., 2017).

Saxagliptin prevents Ang II from activating a variety of cardiac pro-inflammatory/profibrotic signaling intermediates, e.g., NF-κB and IL-17A. TNF-α has also been positively linked to HTN (Jia et al., 2018). It has been demonstrated that DPP-4i reduces TNF-α expression (Lin and Lin, 2016; Zhang et al., 2019).

OS drives NLRP3 inflammasome activation, which induces NF-κB to promote various inflammatory genes, e.g., IL-1 (Zhang et al., 2021). This causes inflammatory cells to be drawn to the vascular wall, intensifying ROS synthesis and impairing endothelial cells (ECs). ROS enzymes, including the Nox family, were lowered in ECs when DPP-4i was administered (Gao et al., 2020; Fontes et al., 2023), with upregulation of antioxidants such as SOD. In addition, the DPP-4i acts as ROS scavenger (Salheen et al., 2015; Kim et al., 2018). Saxagliptin substantially decreased serum levels of IL-1β and TNF-α. In mice with diabetes mellitus, it decreased the activation of NLRP3, as well as the renal production of TNF-α and caspase 1 (Birnbaum et al., 2016).

Histologically, administration of Sita in the HN + Sita group led to a noticeable improvement in kidney structure on H&E stain. Also, on MT stain examination, there was a marked decrease in fibrous tissue around the corpuscles and in between tubules. Moreover, both inflammatory (TNF-α) and apoptotic (BAX) markers were significantly decreased. Such outcomes were consistent with earlier research (Abdel-Zaher et al., 2022), which noticed improvement of the histological structure of DN after administration of Sita. This could be explained by the significant increase in serum stroma-derived factor1-α, which induced the formation of new blood vessels and increased renal endothelial NOS (eNOS), resulting in decreased nephropathy (Ahmed et al., 2016).

### 4.4 Sitagliptin’s superior renoprotective efficacy vs. canagliflozin

Despite both treatment regimens effectively alleviating HN, noticeably, Sita exhibited a better profile in reducing SBP and maintaining baseline microvascular architecture than Cana. Severe systemic endothelial impairment resulting from long-term suppression of NOS is the primary pathophysiology in an L-NAME-induced HTN paradigm (Vazifeshenas-Darmiyan et al., 2026). Sita’s pharmacological activity, which is the structural stability of endogenous active glucagon-like peptide-1 (GLP-1) by DPP-4 inhibition, is the main cause of the substantial improvement shown with this Sita (Hyder et al., 2025). A growing body of research suggests that stabilized GLP-1 binds directly to GLP-1 receptors on endothelial and vascular SMCs, initiating a downstream signaling cascade through phosphatidylinositol 3-kinase/protein kinase B and cyclic adenosine monophosphate/protein kinase A pathways (Hassan et al., 2025). This activation directly promotes the phosphorylation and residual upregulation of eNOS, therefore optimizing any remaining ability for regional NO synthesis with consequent vascular relaxation (Abd El Motteleb and Elshazly, 2013; Saraiva and Sposito, 2014; Drucker, 2016). On the other hand, Cana lacks this specific, direct GLP-1-mediated microvascular endothelial recovery axis, although providing remarkable osmotic and hemodynamic decompression through SGLT2 inhibition (Caturano et al., 2025). This significant distinction offers a credible physiological explanation for Sita’s improved vascular function and noticeable architectural and functional renoprotection in a low-NO hypertensive, non-diabetic condition.

### 4.5 Limitations of the study

Even with the noteworthy outcomes, our study still has limitations. First, although the sample size was calculated *a priori*, the study remains exploratory because multiple biochemical, molecular, and histopathological endpoints were assessed; therefore, confirmatory studies with larger cohorts are warranted. Furthermore, the 5-week treatment course might not adequately reflect the cumulative impact of Sita and Cana on HN, requiring longer-term studies. Given the possibility of gender disparities in treatment reactions, the complexity of the disease in humans may not be well represented using a single animal model. Furthermore, even though the study demonstrated notable physiological changes, it did not thoroughly examine all molecular mechanisms behind these results. The findings could potentially be skewed by the lack of control over dietary components and the restricted evaluation of possible long-term drug adverse effects. Also, the absence of Cana-alone and Sita-alone groups, as well as a standard antihypertensive comparator such as an ACEI or ARB, is another limitation. These additional controls could help in distinguishing drug-specific from broader systemic effects and could strengthen the mechanistic interpretation of Ang II-related signaling. Moreover, RAGE and Nox4 were evaluated at the mRNA level only; therefore, the proposed involvement of the Ang II/RAGE/Nox4/NLRP3 pathway should be interpreted cautiously until confirmed by protein-level studies such as Western blotting. Furthermore, because our experimental design did not utilize site-specific molecular antagonists or genetic knockout models (such as RAGE^−/−^ or NLRP3^−/−^ vectors), a definitive, direct causal relationship linking the individual nodes of this cascade cannot be unequivocally proven. The observed renoprotection represents a comprehensive systemic and intrarenal therapeutic outcome; therefore, subsequent investigations using targeted molecular gene silencing or selective antagonists are warranted to dissect the exact node-specific dependencies of Cana and Sita in HN. Therefore, these restrictions show that more research is necessary to confirm and elaborate on these results in a therapeutic setting and our findings should be interpreted as preclinical and hypothesis-generating.

### 4.6 Recommendations and perspectives

Future studies should scrutinize the chronic side effects of Cana and Sita using larger sample sizes of varied animal models, including both genders. To independently prove such conclusions and assess these medications’ efficacy and safety in patients with HN, human clinical studies are crucial and Western blot methods should be utilized to confirm transcriptional results.

## 5 Conclusion

In this short-term rat model, Cana and Sita demonstrated significant protective effects on HN. Both treatments effectively reduced SBP and improved renal functions. These effects may be associated with the modulation of Ang II/RAGE/Nox4/NLRP3-related inflammatory and oxidative pathways. Within the limits of this study, Sita showed more favorable outcomes compared to Cana on some of the measured parameters, which necessitates further investigation in clinical settings. While these findings support further preclinical investigation, they do not yet establish clinical efficacy or superiority in humans.

## Data Availability

The original contributions presented in the study are included in the article/supplementary material, further inquiries can be directed to the corresponding author.
